# Case Report: PRRT in a patient with Zollinger-Ellison syndrome. The management of gastrointestinal complications

**DOI:** 10.3389/fonc.2025.1590478

**Published:** 2025-05-19

**Authors:** Martina Di Franco, Rexhep Durmo, Maria Liberata Di Paolo, Roberto Giacosa, Elisa Bannò, Valentina Ambrosini, Angelina Filice

**Affiliations:** ^1^ Nuclear Medicine, Alma Mater Studiorum, University of Bologna, Bologna, Italy; ^2^ Nuclear Medicine Unit, Azienda Unità Sanitaria Locale-IRCCS di Reggio Emilia, Reggio Emilia, Italy; ^3^ Gastroenterology Unit, Azienda Unità Sanitaria Locale-IRCCS di Reggio Emilia, Reggio Emilia, Italy; ^4^ Oncology Medicine Unit, Area Nord-Mirandola, AUSL Modena, Modena, Italy; ^5^ Nuclear Medicine, IRCCS, Azienda Ospedaliero-Universitaria di Bologna, Bologna, Italy

**Keywords:** gastrointestinal cancers, gastric and esophageal cancers, PRRT, NET, Zollinger-Ellison syndrome

## Abstract

Zollinger-Ellison Syndrome (ZES) is a rare condition characterized by excessive gastric acid secretion due to gastrin-producing neuroendocrine tumors. Peptide Receptor Radionuclide Therapy (PRRT) with [177Lu]Lutetium Oxodotreotide is an effective treatment for advanced neuroendocrine tumors, including those associated with ZES. However, the gastrointestinal toxicity induced by ZES can complicate the administration of PRRT. We present a case of a patient with metastatic G2 neuroendocrine tumor of the duodenum and ampulla of Vater who experienced severe gastrointestinal complications after the first PRRT cycle due to exacerbated ZES. The implementation of a prophylactic treatment with high-dose proton pump inhibitors before and after the subsequent PRRT cycles allowed for the successful completion of the therapy. This case highlights the importance of considering ZES-related complications in patients undergoing PRRT. Proactive management with high dose acid-suppressing therapy can significantly improve patient tolerance and treatment outcomes. Further research is needed to optimize the management of ZES patients undergoing PRRT.

## 1 Introduction

Zollinger-Ellison Syndrome (ZES) is a rare disorder characterized by excessive acid production due to gastrin-secreting neuroendocrine tumors (NET), which typically originate in the pancreas, duodenum or extra-hepatic biliary system (the “gastrinoma triangle”) (1). Symptoms include gastro- esophageal reflux, diarrhea, abdominal pain and anemia due to the presence of peptic ulcers (2, 3). The acid hypersecretory state can be controlled either through surgical removal of the primary tumor and/or medication therapy with gastric acid suppressant drugs. Despite symptoms control, 60-90% of patients with gastrinomas develop metastatic lesions, mostly in the liver, and ultimately require further therapies (4, 5).

Peptide Receptor Radionuclide Therapy (PRRT) with [177Lu]Lutetium Oxodotreotide is approved for unresectable or metastatic, progressive, well-differentiated (G1 and G2), SST-positive gastro-entero-pancreatic NET, including ZES-associated tumors (6–8). Although PRRT demonstrated to be effective in treating advanced gastrinomas (9, 10) the gastrointestinal toxicity caused by ZES can pose significant challenges to the successful application of PRRT.

We present the case of a patient diagnosed with metastatic G2 NET, who developed severe complications linked to ZES after the first PRRT cycle.

## 2 Case description

**Figure 1:**
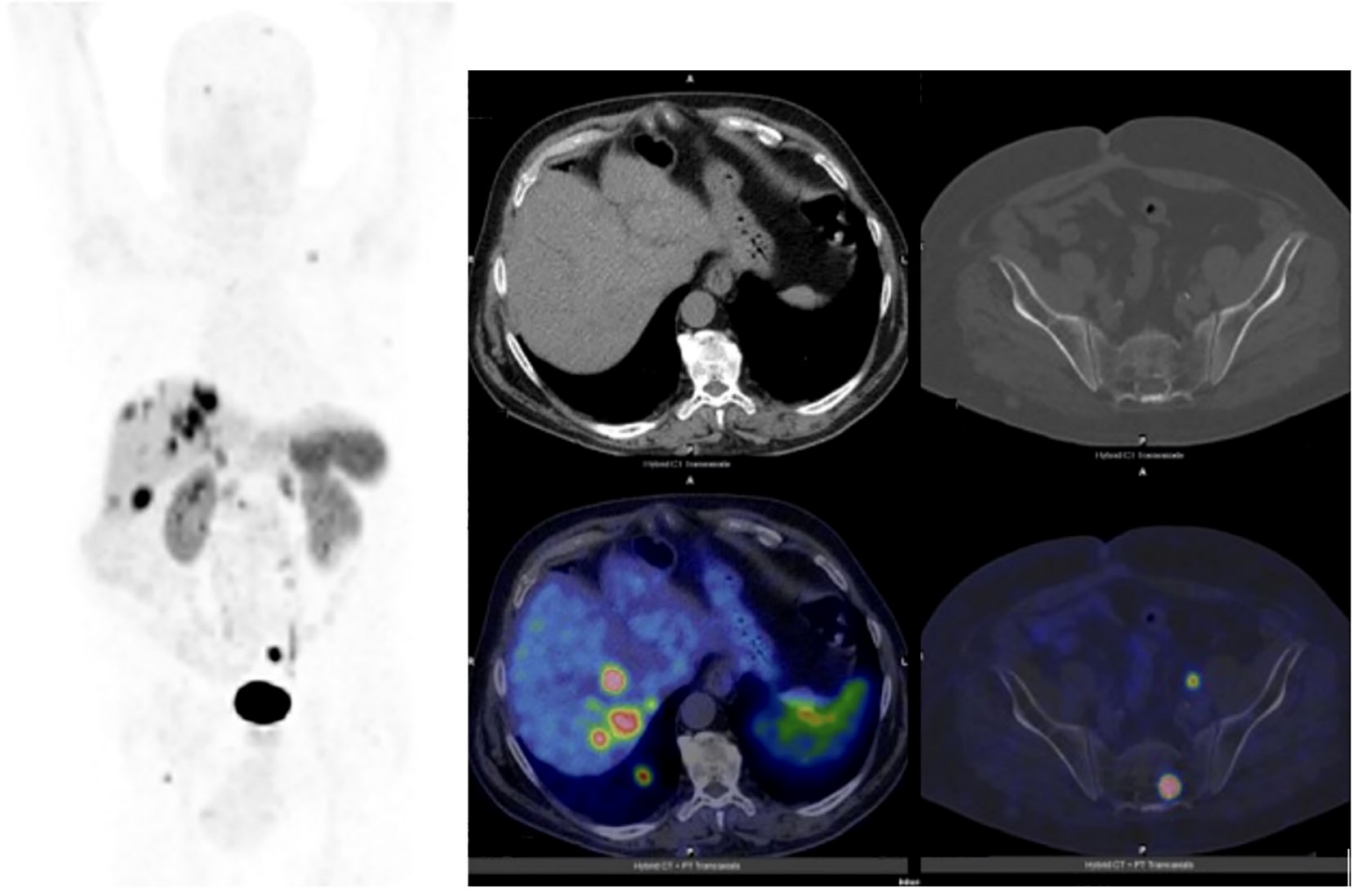
Maximum-intensity-projection and transaxial images of a the [68Ga]Ga-DOTATOC PET/CT performed in March 2023 for restaging after somatostatin analogues initiation. The PET scan demonstrated disease progression due to the appearance of SST uptake in multiple secondary hepatic and bone lesions. 125.

In September 2021, a 72-year-old patient presenting with vomiting, diarrhea and dyspepsia was diagnosed with a duodenal NET, following the endoscopic identification and biopsy of a duodenal lesion, accompanied by multiple duodenal ulcers. Staging with contrast-enhanced computed tomography demonstrated one duodenal lesion only and no evidence of metastatic disease. The diagnosis was confirmed as a Grade 2 (G2) NET with a Ki-67 proliferation index of 5% after the patient underwent a laparotomic segmental duodenal resection with curative intent. Pantoprazole 40 mg daily was prescribed as home therapy at the time of diagnosis. In November 2021, he was hospitalized due to severe anemia and received endoscopic treatment of a bleeding esophageal ulcerative lesion. In January 2022, [68Ga]Ga-DOTATOC PET/CT scan revealed multiple secondary hepatic lesions and two additional uptake areas corresponding to a pancreatic and a duodenal lesion. The duodenal lesion was sampled for histopathological analysis, which confirmed the diagnosis of a second NET of the ampulla of Vater. Therefore, in February 2022, somatostatin analogue (SSA) therapy (Lanreotide 120 mg) was initiated. A sequential assessment performed in March 2023 through [68Ga]Ga-DOTATOC PET/CT demonstrated the appearance of SST uptake in multiple secondary hepatic and bone lesions (**Figure 1**).

**Figure 2:**
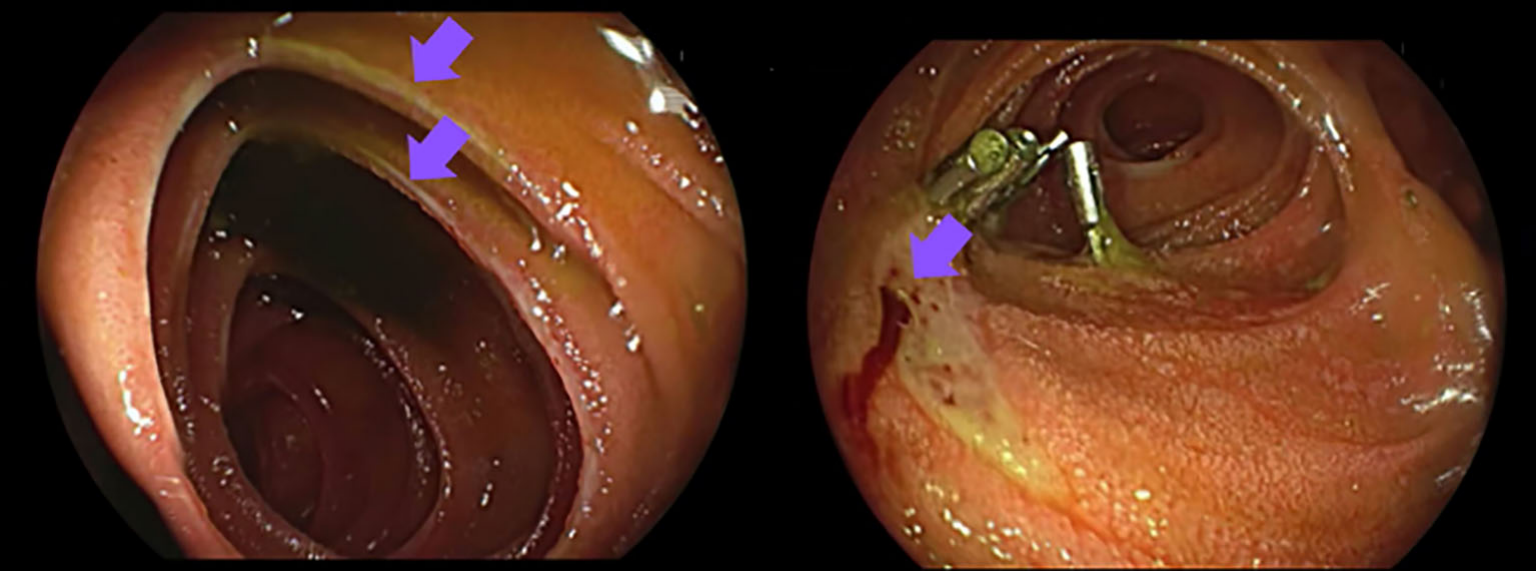
In June 2023 the patient was hospitalized due to hematemesis. An esophagogastroduodenoscopy showed erosive ulcerative esophagitis with duodenal ulcers, requiring mechanical and injective hemostatic interventions. 129.

After multi-disciplinary discussion, due to the evident disease progression, the patient was selected for PRRT with [177Lu]Lutetium Oxodotreotide (Luthathera^®^, Advanced Accelerator Applications Italy S.r.l) according to the standard protocol of four cycles with an activity of 7.4 GBq per cycle. Therapy with SSA was continued and oral pantoprazole 40 mg therapy was also maintained. Following the administration of the first PRRT cycle on May 24^th^, 2023, the patient experienced abdominal pain and incoercible vomiting. Computed tomography and esophagogastroduodenoscopy revealed distal esophageal abnormalities, i.e. circumferential thickening, edema and hyperemia of the walls, with blood traces present. In June 2023, the patient experienced an additional episode of hematemesis that required hemostatic interventions (mechanical and injective), leading to clinical stabilization (**Figure 2**).

## 3 Diagnostic assessment and treatment

The initial detection of elevated serum gastrin levels—1650 pg/ml in October 2021—supported the suspicion of ZES. Following therapeutic interventions, including SSA administration, gastrin levels decreased and measured 764 pg/ml prior to the first administration of [177Lu]Lutetium Oxodotreotide. Due to the complications occurred after the first cycle, a multidisciplinary discussion was performed to decide whether to continue PRRT or not. After a consultation among nuclear medicine physicians, oncologists, and clinicians, it was decided to proceed with a second PRRT cycle, establishing a concomitant acid-suppressing therapy.

**Figure 3:**
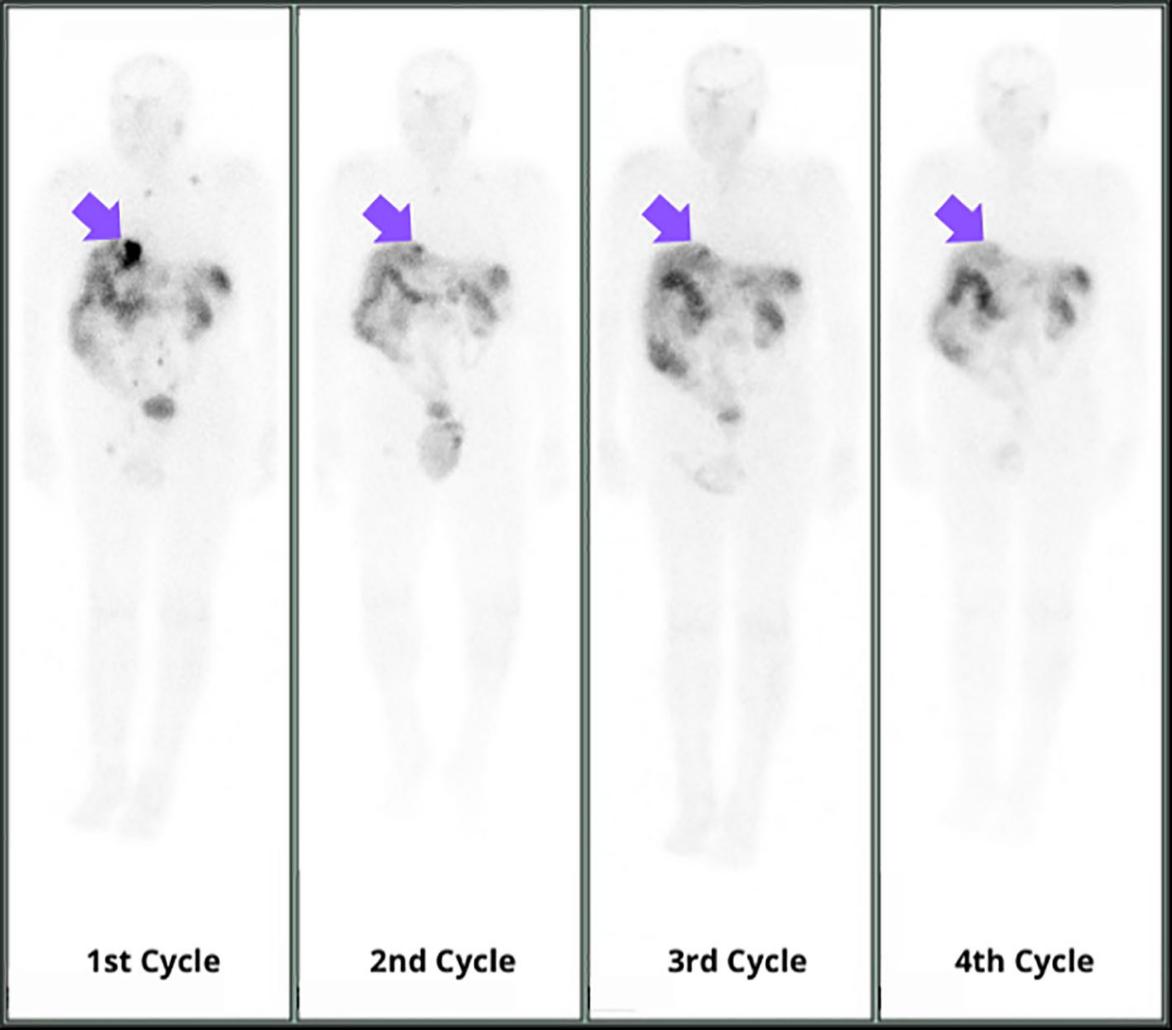
Post-therapy whole-body scans following the four PRRT cycles revealed a notable reduction in size of the tumor lesions, indicating a favorable response to the treatment. 133.

**Figure 4:**
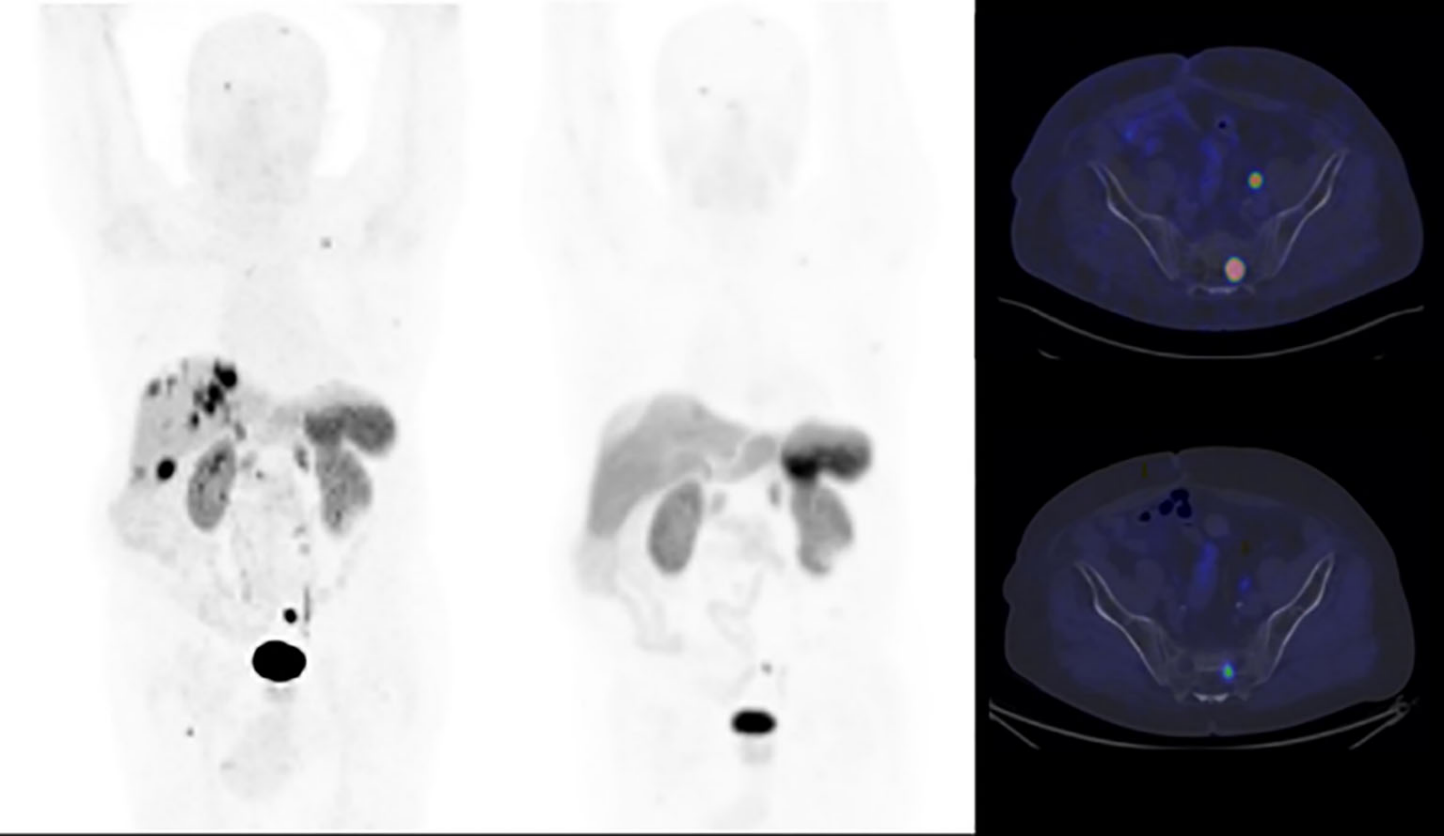
Maximum-intensity projections (MIP) and transaxial images of baseline and end-of-treatment PET/CT scans with [68Ga]Ga-DOTATOC. The end-of-treatment PET/CT confirmed a reduction in number and size of tumor lesions, compared with the baseline PET scan. A partial response was obtained. 139.

Accordingly, immediately after the admission for the second PRRT cycle in July 2023, the patient underwent preparatory treatment with proton pump inhibitors (continuous infusion of physiological solution 250 mg + omeprazen 200 mg at 11 ml/h). This regimen was also maintained after the administration of [177Lu]Lutetium Oxodotreotide, until discharge. No gastrointestinal symptoms were registered during the three-days hospitalization, and subsequent endoscopic assessments did not reveal esophageal or duodenal edema or active bleeding. Due to these results, it was decided to continue this medication regimen for the remaining 2 cycles. The post-therapy whole-body scan following the subsequent cycles revealed a notable reduction of the tumor lesions’size, indicating a favorable response to the treatment (**Figure 3**). The partial response obtained was confirmed by both [68Ga]Ga- DOTATOC PET/CT and contrast-enhanced CT post-therapy assessments (**Figure 4**). Symptomatic relief was also achieved, along with a consistent reduction in gastrin levels over the course of the radioligand treatment (171 pg/ml in November 2023).

## 4 Discussion

Gastrinomas have been reported to be responsive to PRRT in terms of disease control and symptoms mitigation (9, 10). In fact, although the use of proton pump inhibitors (PPIs) can be considered a cornerstone for the pharmacotherapy of ZES and can successfully control the symptoms alone, PRRT can determine a synergistic effect on reducing the hypersecretory state. In the study by Grozinsky- Glasberg et al, PRRT performed in 11 patients with gastrinoma lead to symptomatic improvement (with a decrease in mean serum gastrin in 81% cases), favorable response (complete response in 9%, partial response in 45%, stable disease in 45%), and outcomes (median PFS of 14 months in 64%) (10).

In another study, PRRT performed in 36 patients resulted in morphological, biochemical or clinical response in 26/36 patients and a mean OS of 45.1 months in responders (9). It is known that PRRT can exacerbate symptoms in patients with functioning NET by both causing tumor lysis and hormones release and inducing an inflammatory state that can determine a worsening of the existing gastrointestinal issues (11). In patients with ZES, this can translate in a transient increase in gastrin levels and in a mucosal damage, potentially leading to severe complications such as bleeding ulcers. Nausea and vomiting can be both ZES symptoms and side effects of PRRT, as they have been described alongside the amino acid infusion that precedes the administration of the 177Lu-labeled somatostatin analogue (6). In these patients, gastrointestinal toxicity can significantly impact the clinical outcome.

ENETS guidelines recommend adequately high doses of PPIs during surgery and 3 months afterward in patients with gastrinomas, due to the high risk of gastrointestinal perforation and hemorrhage. Initial PPIs doses should be high (e.g. 60–80 mg/day of omeprazole), with subsequent adjustments made according to the patient’s symptom response. In acute settings, intravenous formulations are recommended (12).

In our case, while oral home therapy with 40 mg pantoprazole was sufficient to control symptoms prior to PRRT, the use of intravenous high dose PPIs as preparation and during each PRRT administration was crucial for the successful mitigation of symptoms throughout the entire treatment course. This highlights the need to investigate the presence of peptic disease, sporadic or due to ZES, in patients with duodenal or pancreatic NET undergoing PRRT. Accurate diagnostic assessments to detect peptic disease can play a crucial role in avoiding severe complications, further hospitalizations and, ultimately, PRRT interruption.

In conclusion, when ZES is suspected or diagnosed, specific preparatory measures, such as high dose proton pump inhibitor infusion, should be taken in order to mitigate potential toxicity. These measures, together with close monitoring of the potential side effects, can enhance patient tolerance and improve treatment success. Further research is warranted to establish optimal strategies for preventing and managing gastrointestinal toxicity in ZES patients undergoing PRRT.

## Data Availability

The original contributions presented in the study are included in the article/supplementary material. Further inquiries can be directed to the corresponding author.
